# Maximal Handgrip Strength as a Measure of General Functional Capacity Rather than Parkinson’s Disease-Specific Impairment

**DOI:** 10.3390/brainsci16080871

**Published:** 2026-08-17

**Authors:** Eduardo Villamil-Cabello, Elvira Molinero-Martín, Noa Fogelson, Antonio Luque-Casado, Miguel Ángel Fernández-del-Olmo

**Affiliations:** Center for Research in Sport Sciences, Rey Juan Carlos University, Camino del Molino s/n, 28943 Fuenlabrada, Madrid, Spain; eduardo.villamil@urjc.es (E.V.-C.); elvira.molinero@urjc.es (E.M.-M.); noa.fogelson@urjc.es (N.F.); antonio.luque@urjc.es (A.L.-C.)

**Keywords:** Parkinson’s disease, handgrip strength, gait, rate-of-force development, functional capacity, older adults, physical function

## Abstract

**Highlights:**

**What are the main findings?**

- Peak grip force was associated with gait speed and step length in both Parkinson’s disease and healthy control groups.
- Upper-limb rate-of-force development and impulse were not associated with gait performance, while participants with Parkinson’s disease showed poorer gait performance than healthy controls.

**What are the implications of the main findings?**

- Maximal grip strength appears to reflect general functional capacity rather than a Parkinson’s disease-specific impairment.
- Handgrip strength may serve as a practical clinical indicator of mobility-related function.

**Abstract:**

**Background/Objectives:** Gait impairment and reduced handgrip strength are common in Parkinson’s disease (PD), but their relationship remains unclear. This study examined whether associations between handgrip force and gait reflect PD-specific impairment or broader functional capacity. **Methods:** In this cross-sectional study, 45 individuals with PD and 51 healthy older adults completed unilateral maximal isometric handgrip testing and overground gait assessment at preferred and maximal speed. Handgrip variables included peak force (PF), rate-of-force development (RFD), and impulse. Gait speed, cadence, and step length were recorded. **Results:** PF was significantly associated with gait speed and step length in both groups, especially under maximal-speed conditions. RFD and impulse were not significantly associated with gait performance in either group. Compared to controls, participants with PD showed poorer gait performance, with lower preferred walking speed and shorter step length in both gait conditions. Within the PD group, PF was lower on the more-affected side than on the less-affected side, whereas most RFD and impulse variables showed no side differences. **Conclusions:** Maximal handgrip strength, but not upper-limb RFD or impulse, was associated with key gait outcomes in both PD and healthy older adults. These findings suggest that handgrip strength may reflect general functional capacity rather than a PD-specific neuromuscular mechanism and support its use as a practical clinical indicator of mobility-related function.

## 1. Introduction

Parkinson’s disease (PD) is a progressive neurodegenerative disorder characterized by bradykinesia, rigidity, tremor, and postural instability [1]. These motor symptoms impair both gait and upper-limb function, including handgrip strength [2,3], which are two domains that are essential for daily functioning and quality of life. Better understanding of the relationship between gait performance and handgrip-force production in individuals with PD may help refine clinical assessment and identify relevant targets for rehabilitation.

Gait impairment is a core feature of PD. Compared to healthy adults, individuals with PD typically show reduced preferred walking speed, stride length, swing time, and hip excursion [4,5]. These alterations contribute to the characteristic shuffling gait and are associated with impaired mobility and increased fall risk. In addition, many individuals experience difficulties with gait initiation and maintenance, including episodes of freezing of gait (FOG), which further compromise functional independence [6]. Prospective evidence also suggests that slower walking pace is associated with a higher risk of incident PD [7], supporting the view that gait dysfunction may reflect clinically relevant aspects of the neurodegenerative process.

Handgrip strength has also emerged as a clinically relevant marker in PD [3,8]. Reduced grip strength has been associated with altered muscle activity and functional decline [2,8], and lower strength has been linked to a higher incidence of PD, particularly in individuals with greater genetic susceptibility [9]. Moreover, handgrip strength has been identified as a predictor of disease severity, especially in patients with FOG [10]. Sarcopenia, which appears to be more pronounced in individuals with PD, may further contribute to both reduced grip strength and impaired gait performance [11]. Together, these findings support the clinical utility of handgrip assessment while also raising the question of what aspect of motor dysfunction it reflects in PD.

Beyond maximal force, the rate-of-force development (RFD) may provide additional insight into neuromuscular impairment. In healthy neuromotor systems, rapid isometric contractions generally maintain a relatively consistent time-to-peak force across force magnitudes through the modulation of RFD [12,13]. Although RFD declines with age, this timing strategy appears to remain largely preserved in older adults [14]. In contrast, individuals with PD show reduced RFD and rate-of-force relaxation compared to age-matched controls [15,16,17] and may require longer muscle activation pulses to generate high peak forces during rapid contractions [16,17]. Because RFD reflects the ability to generate force quickly, it may be particularly sensitive to neuromuscular changes associated with neurodegeneration [14]. However, most evidence in PD comes from lower-limb assessments [14,18], whereas upper-limb RFD remains comparatively underexplored. This is clinically relevant because rapid grip-force generation is important for many activities of daily living [19], and strength training has been shown to improve both RFD and functional performance in PD [20].

Although gait impairment and reduced handgrip force are both common in PD, their interrelationship remains insufficiently understood. These deficits may share common underlying mechanisms, including dopaminergic dysfunction, impaired motor planning, and altered cortico-subcortical motor network dynamics [21,22]. Some studies have reported associations between weaker grip strength and poorer gait performance or greater gait variability in PD [23], suggesting that upper- and lower-limb impairments may reflect related aspects of motor system deterioration. However, evidence from healthy older adults is mixed. While handgrip strength has been associated with gait speed and functional mobility [23,24], and some studies suggest that RFD may be a stronger predictor of mobility than maximal strength [18], findings remain mixed. It therefore remains unclear whether the relationship between handgrip-force production and gait in PD reflects a disease-specific neurodegenerative process or a broader association between general functional capacity and mobility that is also present in healthy aging.

Accordingly, the primary aim of this study was to investigate the relationship between handgrip strength parameters, including peak force and RFD, and spatiotemporal gait parameters in individuals with PD and healthy controls, and to determine whether these associations differ between groups. A secondary aim was to compare handgrip strength parameters between groups while accounting for clinical lateralization in PD (more-affected vs. less-affected side) and hand dominance in healthy controls (dominant vs. non-dominant hand).

## 2. Materials and Methods

### 2.1. Participants

The sample comprised 45 individuals with Parkinson’s disease (PD) and 51 community-dwelling healthy controls (HC), matched for age and sex. Participants were recruited at the Motor Control Laboratory, Rey Juan Carlos University, Madrid (Spain), between September 2022 and May 2023. Table 1 shows the clinical characteristics of Parkinson’s patients.

Inclusion criteria for the PD group were (1) idiopathic Parkinson’s disease diagnosed by a movement disorder specialist according to the UK Parkinson’s Disease Society Brain Bank criteria; (2) Hoehn and Yahr stage 1–3; (3) age ≥ 40 years; and (4) ability to walk independently without assistive devices for at least 10 m. Exclusion criteria for both groups were atypical or secondary parkinsonism, other neurological disorders (e.g., stroke, dementia, or peripheral neuropathy), severe musculoskeletal conditions affecting gait or upper-limb function, surgery within the previous 6 months, unstable cardiovascular or respiratory disease, and cognitive impairment that precluded informed consent or comprehension of the testing procedures.

All participants were informed about the purpose of the study and provided written informed consent before testing. The study was registered at ClinicalTrials.gov (Identifier: NCT05829915), conducted in accordance with the Declaration of Helsinki, and approved by the Ethics Committee of Rey Juan Carlos University (reference 201120245922024, date 19 February 2020).

### 2.2. Study Design and Procedure

A cross-sectional observational study was conducted. The experimental protocol consisted of a single laboratory session divided into two phases and lasted approximately 20 min. Participants in the PD group had been on a stable dopaminergic medication regimen for at least 1 month and were assessed in the practically defined ON-medication state, typically 1–2 h after medication intake, to reflect their usual daily motor functioning. ON status was confirmed by both the participant and the clinical evaluator before testing. All participants were instructed to avoid strenuous physical exercise during the 24 h preceding the assessment.

In the first phase, motor disability in the PD group was assessed using the motor section of the Movement Disorder Society—Unified Parkinson’s Disease Rating Scale (MDS-UPDRS part III) [25] and the Hoehn and Yahr scale [1,26]. Anthropometric characteristics (body mass and height) were then measured in all participants. Finally, hand dominance and, in the PD group, clinical asymmetry were determined to establish the order of the subsequent motor assessments.

Hand dominance was defined as the self-reported writing hand, consistent with UK Biobank procedures [27,28], and was additionally assessed using the Edinburgh Handedness Inventory [29]. In the PD group, the more-affected body side was identified on the basis of lateralized motor symptoms derived from MDS-UPDRS part III limb items (rigidity, bradykinesia, and tremor scored separately for the right and left sides) and confirmed by the examining neurologist. Specifically, the side with the higher aggregate motor score was classified as the more-affected side, and the contralateral side as the less-affected side. In cases of symmetrical presentation, the participant-reported side of symptom onset was used for classification.

In the second phase, participants completed the upper- and lower-limb motor assessments. First, they performed the handgrip strength protocol, consisting of unilateral maximal isometric voluntary contractions (MIVCs) using a custom handgrip dynamometer. They then completed the gait assessment.

### 2.3. Handgrip Strength Assessment

Handgrip strength was assessed with a custom-made hand dynamometer using a commercial strain gauge (NL63, 200 kg; Digitimer LTD, Hertfordshire, UK) with the distance between handles adjusted to each participant (50% of the distance between the middle fingertip to the metacarpophalangeal flexion crease at the base of the thumb) [27,28]. The force signal was amplified (×484) and sampled (2 kHz) for offline analysis (Power 1401, Cambridge Electronic Design, Cambridge, UK; sampling frequency 2 kHz). Assessments were performed with participants standing upright, feet flat on the floor, and hips and knees fully extended. The tested arm was positioned with the shoulder in neutral (0° flexion and abduction), the elbow fully extended, and the forearm in a neutral position.

Before testing, participants were familiarized with the dynamometer and performed one submaximal familiarization trial. They then completed two MIVCs with each hand. In the HC group, testing began with the dominant hand, whereas in the PD group, it began with the more-affected hand. Sides were alternated between trials. Each MIVC lasted up to 5 s. Standardized verbal instructions were provided before each trial to encourage maximal and rapid force production (“squeeze the dynamometer as hard and as fast as possible”). A 60 s rest period was provided between trials to minimize fatigue [30].

**Figure 1:**
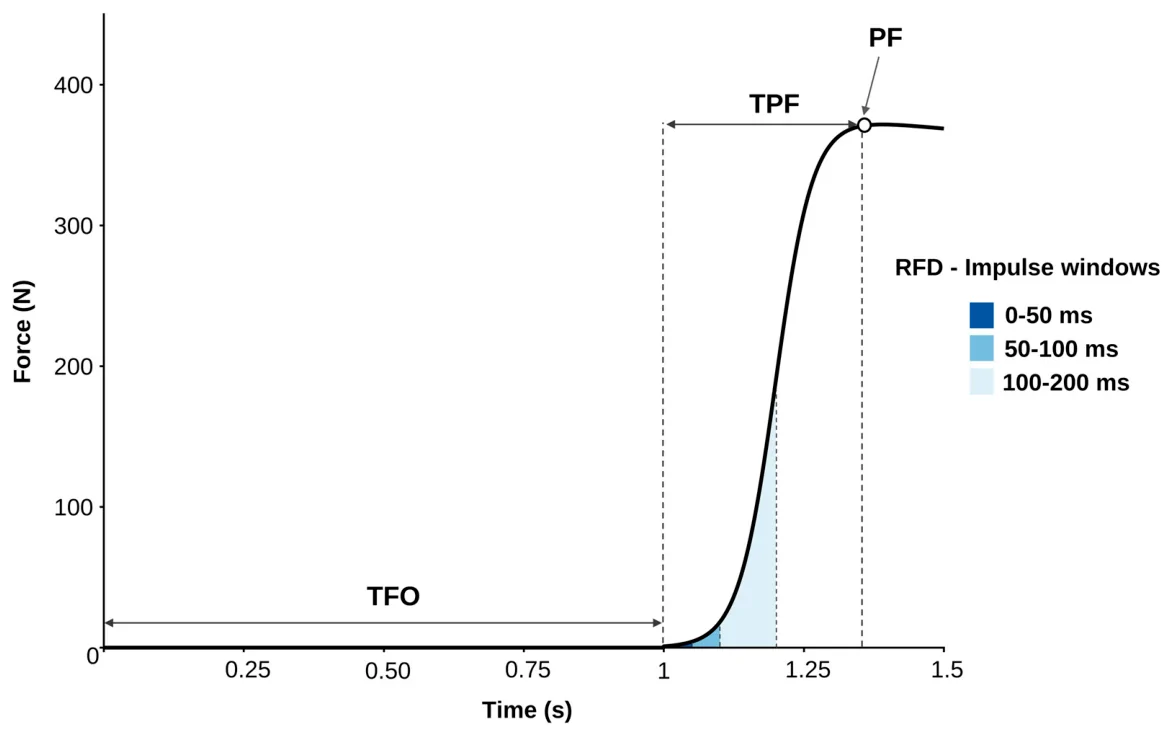
Force–time curve. TFO, time-to–force onset; TPF, time–to–peak force; PF, peak force; RFD, rate–of–force development.

Force was recorded in Newtons (N). For each hand, the trial with the highest force value was used to determine maximal handgrip strength, defined as peak force (PF) [27,28]. From the force–time curve of that trial, the following variables were extracted (see Figure 1): time from the start cue to force onset (time-to-force onset, TFO), time from force onset to peak force (time-to-peak force, TPF), rate-of-force development (RFD), and force impulse (Imp; area under the force–time curve). RFD and impulse were calculated over three time windows from force onset, namely 0–50 ms (RFD0–50 and Imp0–50), 50–100 ms (RFD50–100 and Imp50–100), and 100–200 ms (RFD100–200 and Imp100–200), and expressed in N/s and N·s, respectively.

Contraction onset was identified using an automatic threshold-based procedure, defined as the instant at which force exceeded a preset threshold above baseline [31]. Trials were excluded when baseline force was unstable, participants exhibited pre-tension before the start cue, failed to maintain the required testing position, did not produce a clear peak force, or showed an irregular force–time trace.

### 2.4. Gait Assessment

Gait was assessed under two conditions administered in the following order: (1) preferred speed and (2) maximal speed. In both conditions, participants walked overground for 1 min without assistance or obstacles. Standardized verbal instructions were provided for each condition: “walk at your normal, comfortable pace” for the preferred-speed condition and “walk at the maximum possible speed” for the maximal-speed condition.

Spatiotemporal gait parameters were recorded using an optical detection system (OptoGait, Microgate, Bolzano, Italy), which provides reliable gait measurements [32,33]. The system consisted of transmitting and receiving bars with 100 infrared LEDs arranged in parallel along a 5 m active capture area and sampled at 1000 Hz. The full walkway length was 5 m, with sufficient space before and after the active area to allow steady-state walking.

Testing was performed in a quiet, well-lit corridor. For each condition, participants walked continuously back and forth along the walkway for 1 min. In the preferred-speed condition, participants were instructed to walk at their usual comfortable pace. In the maximal-speed condition, they were instructed to walk as fast as possible while maintaining safety. A rest period of at least 60 s was provided between conditions to minimize fatigue.

The following spatiotemporal gait parameters were automatically computed by the OptoGait software (1.14.11) and averaged across the 1 min walking period: speed (m/s), cadence (steps/min), and step length (m), under both preferred- and maximal-speed conditions. The preferred-speed condition was intended to reflect habitual gait performance [34,35], whereas the maximal-speed condition was used to capture gait reserve capacity, which is particularly relevant in PD, where gait slowing is a cardinal motor feature [5,36].

### 2.5. Statistical Analysis

All statistical analyses were performed using JASP software (version 0.19.3) [37]. Mean values and standard deviations were computed for each variable. Normality and equality of variances were assessed using the Shapiro–Wilk and Levene’s tests, respectively, applying non-parametric alternatives where necessary.

First, to ensure comparability between groups and evaluate baseline differences in anthropometric characteristics and absolute maximum force, independent-sample *t*-tests were conducted. Specifically, age, body mass, height, and maximal handgrip strength (PF) for both the dominant and non-dominant hands were compared between the PD and HC groups.

To assess the effect of lateral dominance in the HC group and disease asymmetry in the PD group on grip strength parameters, intra-group comparisons were performed. Specifically, paired samples *t*-tests were used to compare PF between the dominant and non-dominant hands within the HC group, as well as between the clinically more-affected and less-affected sides within the PD group. For non-parametric force variables (i.e., RFD and force impulse parameters), the Wilcoxon signed-rank test was applied.

Additionally, to examine disease-specific strength deficits, independent-sample *t*-tests were conducted to compare the force parameters of the more-affected and less-affected sides of the PD group against the dominant hand of healthy adults. For non-parametric force variables, the alternative non-parametric Mann–Whitney U test was computed.

To evaluate differences in spatiotemporal gait parameters between groups, independent-sample *t*-tests were conducted for speed, cadence, and step length under both walking conditions (preferred and maximal speed).

Finally, to determine whether the relationship between upper-limb force production and lower-limb motor deterioration is disease-specific or a general consequence of reduced functional capacity, correlational analyses were performed. Pearson’s or Spearman’s correlations (depending on data distribution) were used to assess the associations between grip strength parameters and spatiotemporal gait variables within both groups. Furthermore, Spearman’s rank correlation coefficient (rs) was utilized to explore the relationship between clinical disease severity (UPDRS-III) and all force parameters within the PD group.

To account for multiple comparisons and control the Type I error rate, the Bonferroni correction was applied where appropriate. Effect sizes were reported as Cohen’s d for *t*-tests, and Rank–Biserial Correlation (r_rb) for Wilcoxon and Mann–Whitney tests. The level for statistical difference was set at *p* = 0.05.

## 3. Results

The sample characteristics of the participants are presented in Table 2. No significant differences were found between the PD and HC groups regarding age, body mass, or height (*p* > 0.5), confirming that the groups were effectively matched.

Regarding lateralization, 91 out of 96 participants were right-handed (two left-handed in HC; three in PD). In the PD group, disease asymmetry was balanced (21 right-sided; 24 left-sided). The more-affected side corresponded to the dominant hand in 22 patients and the non-dominant hand in 23. Absolute maximal handgrip strength (PF) showed no significant between-group differences for either the dominant (*p* = 0.32) or non-dominant hand (*p* = 0.13).

**Figure 2:**
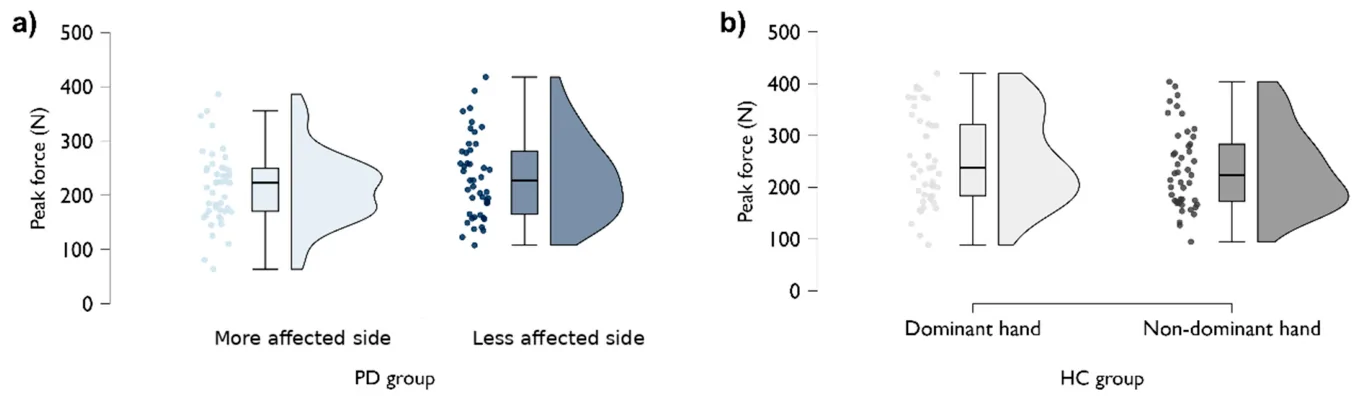
Peak force (PF) for the Parkinson’s disease (PD) and healthy control (HC) groups as a function of side and hand dominance. PF is shown for the more-affected and less-affected sides in the PD group (**a**) and for the dominant and non-dominant hands in the HC group (**b**). Error bars represent 95% confidence intervals.

Regarding intra-group comparisons, paired tests for the PD group revealed that PF was significantly lower on the clinically more-affected side (213.68 ± 67.53 N) than on the less-affected side (232.33 ± 75.27 N), with *t* = −3.37, 95% CI [−29.82, −7.49], *p* = 0.002, and *d* = 0.50 (Figure 2a). Similarly, for the HC group, paired comparisons showed that PF was significantly higher in the dominant hand (251.81 ± 86.53 N) than in the non-dominant hand (234.79 ± 78.01 N), with *t* = 3.6, 95% CI [7.24, 25.74], *p* < 0.001, and *d* = 0.54 (Figure 2b). In contrast, no significant differences were observed between hands and sides for the remaining strength parameters in either group (*p* > 0.05).

**Figure 3:**
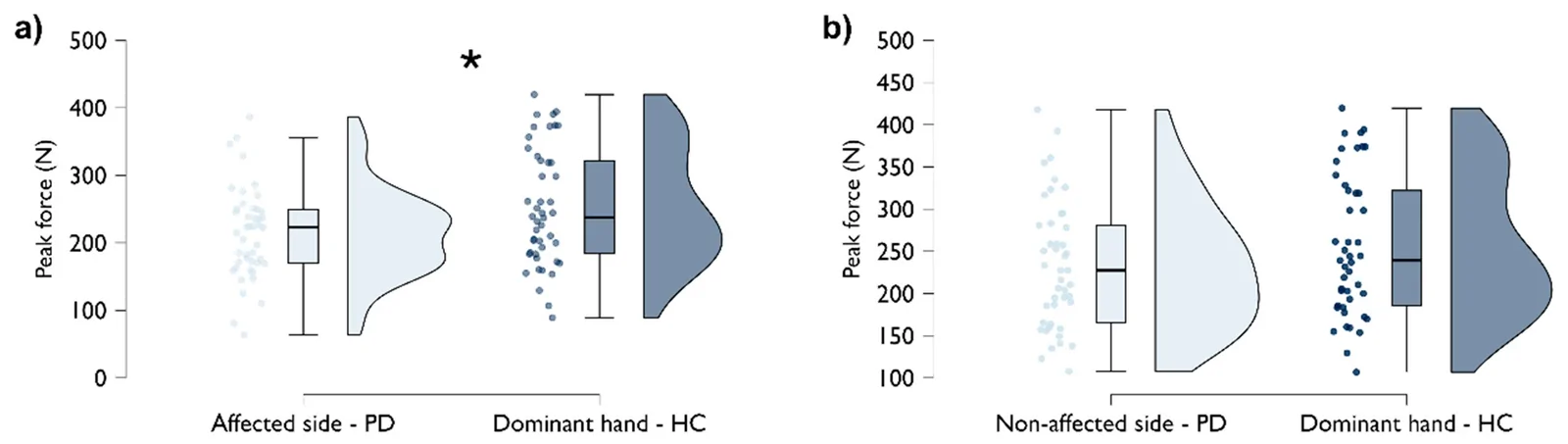
Peak force (PF) comparisons between the Parkinson’s disease (PD) and healthy control (HC) groups. PF is shown for the PD more-affected side versus the HC-dominant hand (**a**) and for the PD less-affected side versus the HC-dominant hand (**b**). Error bars represent 95% confidence intervals. Asterisks indicate significant differences between groups (* *p* < 0.05).

Between-group comparisons revealed that PF on the PD more-affected side was significantly lower than in the HC-dominant hand (*t* = −2.34, 95% CI [−70.51, −5.75], *p* = 0.02, *d* = 0.49; Figure 3a). However, no significant differences were observed when comparing the PD more-affected side with the HC non-dominant hand (*p* > 0.05). Additionally, a significant difference was observed in the RFD 100–200 ms window between the PD more-affected side and the HC-dominant hand (U = 772.0, 95% CI [−439.9, −8], *p* = 0.04, *r_rb_* = 0.25; Figure 3b), with lower values for the PD group. For the PD less-affected side, no significant differences were found compared to either HC hand for any force variable (*p* > 0.05).

Regarding spatiotemporal gait performance, the PD group exhibited significantly lower performance than the HC group in both preferred- and maximal-speed conditions (Table 3). Specifically, after Bonferroni correction (*p* < 0.008), patients with PD showed lower speed (*p* = 0.002, *d* = 0.66) and reduced step length (*p* < 0.001, *d* = 0.81) during the preferred-speed condition, both with large effect sizes. Additionally, in the maximal-speed condition, step length remained significantly shorter in the PD group (*p* = 0.005, *d* = 0.59) with a moderate-to-large effect size. Conversely, speed in the maximal-speed condition (*p* = 0.02) did not meet the adjusted significance threshold. No significant differences were observed for cadence in either the preferred- or maximal-speed condition (*p* = 0.60 and *p* = 0.26, respectively).

To establish the relationship between the level of functional disability (UPDRS-III) and handgrip strength within the PD group, correlation analyses revealed only a significant association between PF on the more-affected side and UPDRS-III scores (rs = 0.34, 95% CI [0.02, 0.61], *p* = 0.04). No significant associations were found for the less-affected side or any other force parameters (*p* > 0.05).

**Figure 4:**
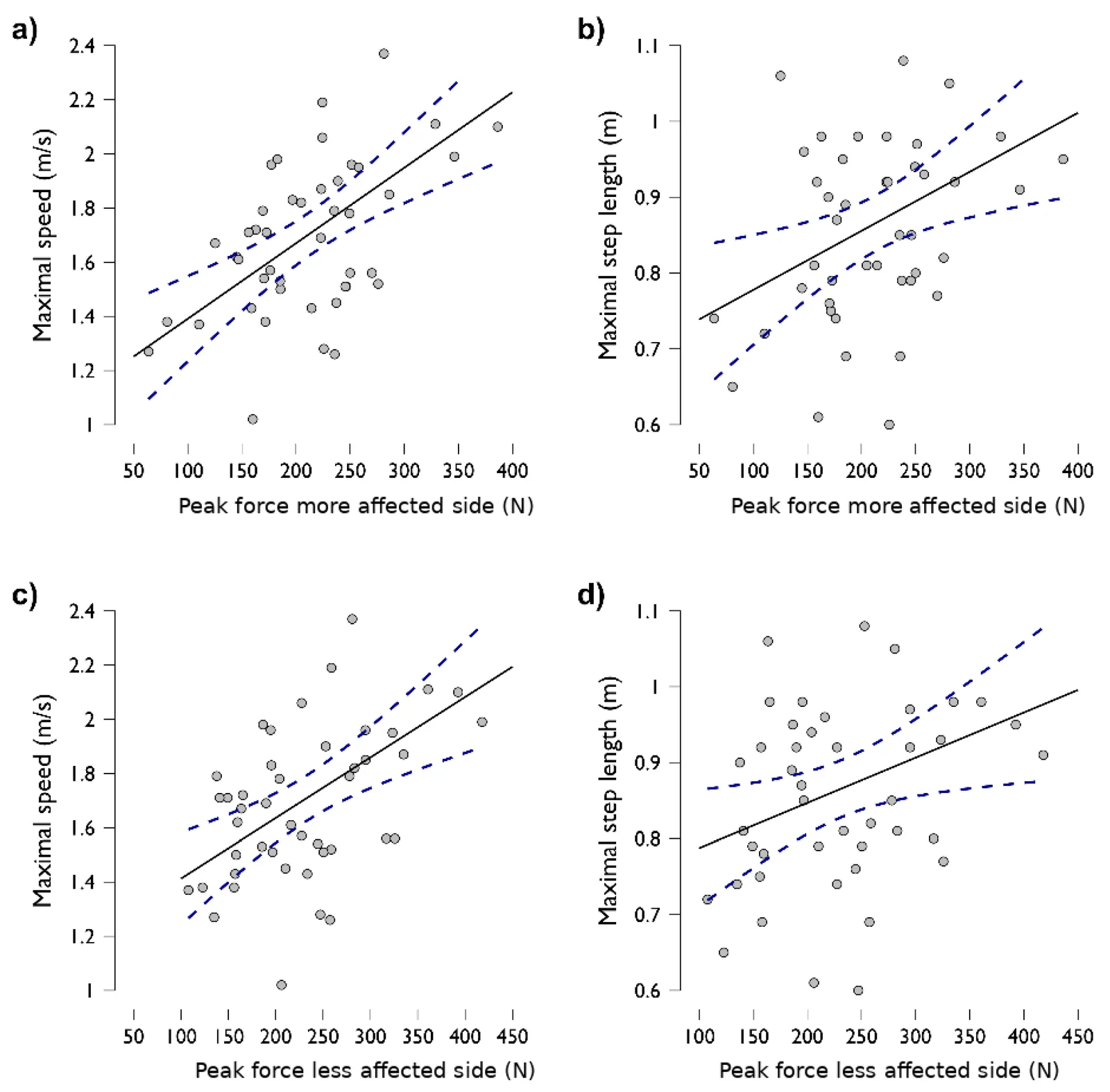
Scatter plots of significant correlations between peak force (PF) and maximal gait parameters in the Parkinson’s disease (PD) group. Data are shown for the handgrip strength of the more-affected side (**a**,**b**) and the less-affected side (**c**,**d**). Black solid lines represent linear regression, and blue dashed lines indicate 95% confidence intervals.

With respect to the relationship between handgrip strength and gait, PF was significantly associated with speed and step length in the PD group in both gait conditions (Figure 4). Specifically, for the more-affected side, significant correlations were found with maximal speed (*r* = 0.58, 95% CI [0.35, 0.75], *p* < 0.001), preferred speed (*r* = 0.38, 95% CI [0.10, 0.60], *p* = 0.01), and step length in the maximal-speed condition (*r* = 0.40, 95% CI [0.12, 0.62], *p* = 0.007). Regarding the less-affected side, PF also correlated significantly with maximal speed (*r* = 0.52, 95% CI [0.27, 0.71], *p* < 0.001), maximal step length (*r* = 0.34, 95% CI [0.05, 0.58], *p* = 0.02), and preferred speed (*r* = 0.30, 95% CI [0.01, 0.55], *p* = 0.046). No significant correlations were found for cadence or any other force variables (*p* > 0.05).

**Figure 5:**
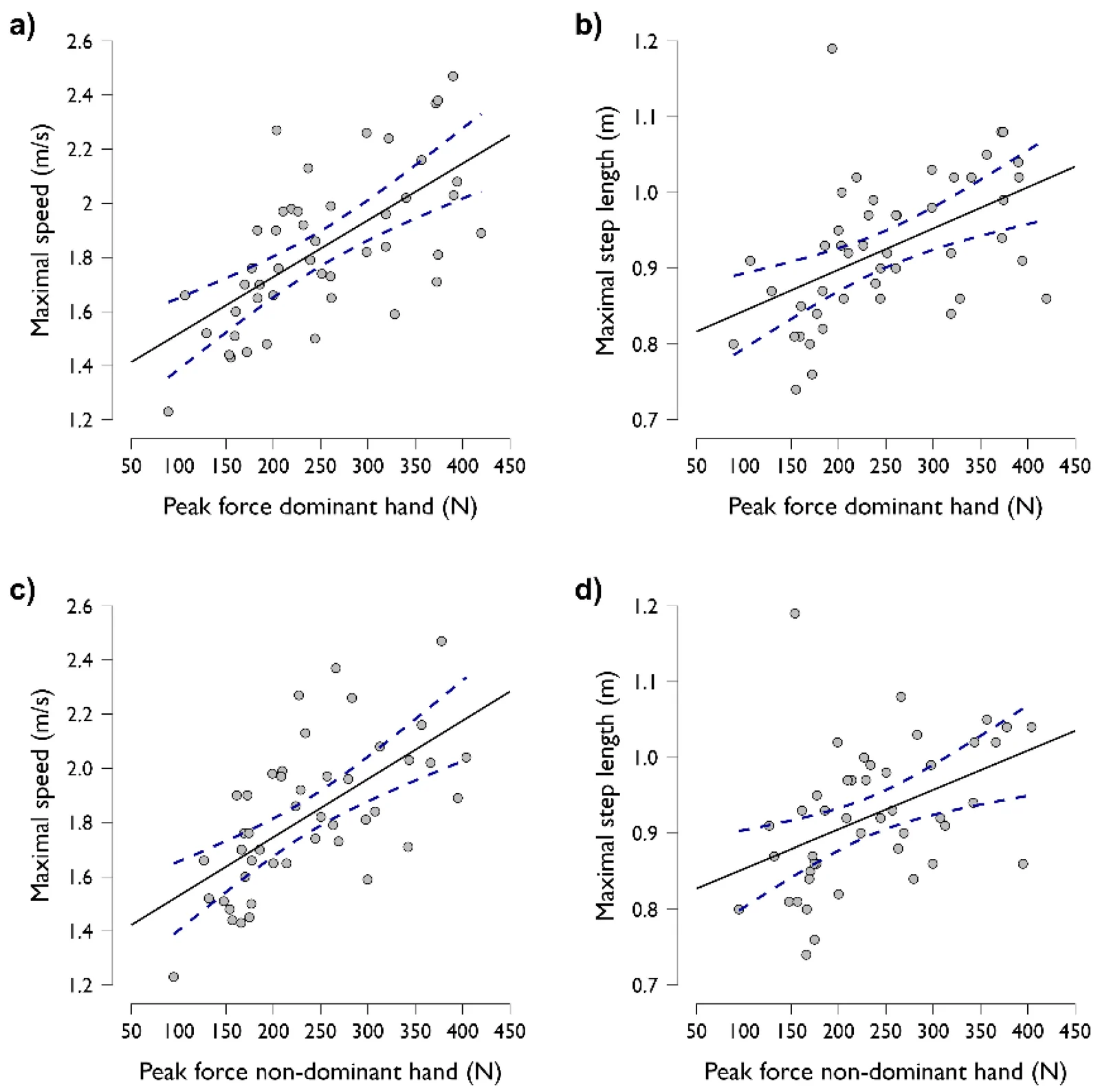
Scatter plots of significant correlations between peak force (PF) and maximal gait parameters in the healthy control (HC) group. Data are shown for the handgrip strength of the dominant hand (**a**,**b**) and the non-dominant hand (**c**,**d**). Black solid lines represent linear regression, and blue dashed lines indicate 95% confidence intervals.

Similarly, in the HC group, PF showed significant positive correlations for both the dominant and non-dominant hands (Figure 5). For the dominant hand, PF correlated significantly with maximal speed (*r* = 0.64, 95% CI [0.44, 0.79], *p* < 0.001), preferred speed (*r* = 0.42, 95% CI [0.15, 0.64], *p* = 0.003), maximal step length (*r* = 0.50, 95% CI [0.25, 0.69], *p* < 0.001), and preferred step length (*r* = 0.39, 95% CI [0.12, 0.61], *p* = 0.007). Finally, for the non-dominant hand, significant associations were observed with maximal speed (*r* = 0.63, 95% CI [0.41, 0.78], *p* < 0.001), preferred speed (*r* = 0.47, 95% CI [0.21, 0.67], *p* = 0.001), maximal step length (*r* = 0.44, 95% CI [0.17, 0.65], *p* = 0.003), and preferred step length (*r* = 0.39, 95% CI [0.11, 0.62], *p* = 0.008).

## 4. Discussion

This study investigated the relationship between handgrip strength parameters and spatiotemporal gait parameters in individuals with PD and healthy older adults. More specifically, it examined whether impaired force production reflects a disease-specific feature or a more general consequence of reduced functional capacity, while also exploring the influence of clinical lateralization in PD and hand dominance in healthy controls (HC) on these associations.

Overall, the findings indicate that peak force (PF) may be a consistent correlate of key gait outcomes, particularly gait speed and step length, in both groups. In contrast, RFD and impulse variables across the different temporal windows were not significantly associated with gait performance in either group. In addition, the PD group showed lower PF as well as reduced gait speed and step length compared to controls, whereas most upper-limb RFD variables were broadly comparable between groups. Taken together, these findings suggest that the association between upper-limb maximal strength and functional mobility may be influenced, at least in part, by general functional capacity rather than by a PD-specific neuromuscular mechanism.

### 4.1. Maximal Grip Strength as a Correlate of Gait Performance

The associations observed between handgrip PF and gait performance, particularly gait speed and step length, in both individuals with PD and HC are consistent with previous studies [10,23]. Prior research has shown that both grip strength and walking pace are associated with incident PD risk [7], suggesting that these parameters may reflect broader physical decline rather than exclusively dopaminergic dysfunction. This interpretation is consistent with the view that grip strength reflects overall musculoskeletal health and general physical conditioning [3,7]. In the present study, the fact that these correlations were observed on both sides in PD and were of similar magnitude to those observed in HC [23,24] further supports the idea that the relationship between upper-limb strength capacity and locomotor performance may remain largely preserved despite neurodegeneration.

The lower gait speed and shorter step length observed in the PD group are also consistent with the established literature [4,36]. These gait impairments are likely multifactorial and may involve dopaminergic deficits affecting basal ganglia motor control [21], as well as peripheral weakness, postural instability, and altered sensorimotor integration [38]. Accordingly, the association between handgrip PF and gait speed in PD may reflect shared reductions in force-generating capacity across muscle groups, suggesting that PF may represent a general functional constraint on mobility in both aging and PD.

### 4.2. Absence of RFD–Gait Associations: Biomechanical and Task-Specific Considerations

In contrast to maximal strength, RFD and impulse variables were not significantly associated with gait parameters in either individuals with PD or healthy older adults. One possible explanation is task specificity. Although lower-limb RFD is biomechanically important for gait performance and balance recovery [18,39], upper-limb RFD may be less directly related to the demands of steady-state locomotion.

In addition, walking at preferred or maximal speed may depend more on overall force-generating capacity than on explosive upper-limb force production. In both PD and healthy aging, gait speed may therefore be more closely linked to total general functional capacity, whereas RFD may become more relevant during reactive or time-constrained tasks, such as rapid postural corrections or fall avoidance [38,39]. From this perspective, the relationship between upper-limb force production and gait may be mediated primarily by overall strength rather than by the rate at which force is generated.

### 4.3. More-Affected Versus Less-Affected Sides

A notable finding of the secondary analysis was the presence of clinical asymmetry in maximal handgrip strength in the PD group. Specifically, individuals with PD showed lower PF on the more-affected side than on the less-affected side, in agreement with the characteristically lateralized presentation of the disease [1]. By contrast, this side-to-side difference was not observed across most RFD and impulse variables, which remained relatively similar between sides. This pattern differs from previous lower-limb studies in which explosive force deficits were often more pronounced than maximal strength impairments [15,16,17,18].

This dissociation between maximal and explosive force was also reflected in the associations with motor symptom severity. Whereas MDS-UPDRS part III scores were significantly associated with PF on the more-affected side, no such associations were observed for RFD or impulse variables. Together, these findings suggest that PF may be a more clinically sensitive marker of motor impairment than explosive force in the context of upper-limb isometric testing.

Several factors may help explain why explosive force appeared less sensitive to clinical lateralization in the present study. One possibility is that upper-limb RFD during a brief isometric handgrip task may be less susceptible to the effects of bradykinesia and rigidity than lower-limb explosive actions, particularly in individuals with mild-to-moderate disease severity [40]. It is also possible that upper-limb RFD deficits become more evident only in advanced disease stages or during more demanding functional tasks, such as rapid finger tapping or precision grip tasks, rather than during maximal isometric handgrip contractions [19]. Another possible explanation is that individuals with PD may rely on compensatory neural strategies, such as prolonged agonist muscle activation, to achieve near-normal explosive output despite underlying neural deficits [16,17]. These interpretations remain speculative, however, and should be confirmed in future studies.

### 4.4. Comparison with Healthy Controls: Maximal Strength Deficits Without Clear RFD Impairment

No broad between-group differences were observed in RFD or impulse variables between the more-affected side of individuals with PD and the dominant side of HC. By contrast, the PD group showed significantly lower PF on the more-affected side than HC. This pattern differs from lower-limb studies that have reported marked impairments in explosive force production in PD [15,16,17,18].

This discrepancy may reflect anatomical and functional differences between upper- and lower-limb motor control in PD. Lower-limb tasks such as gait and postural control rely heavily on motor circuits that are strongly affected by dopaminergic degeneration [21], whereas upper-limb forceful grip tasks may depend to a greater extent on cortical pathways or compensatory motor strategies [22,40]. Methodological differences may also contribute. Handgrip dynamometry assesses isometric force production, whereas lower-limb studies often examine dynamic tasks, which impose different biomechanical and neural demands [18].

Interestingly, a significant difference was observed in the RFD100–200 ms interval between the more-affected side in PD and the dominant hand in HC. This finding may suggest that, although early explosive force production is broadly maintained, individuals with PD may show subtle deficits in sustaining rapid force generation as the contraction progresses. However, this isolated finding should be interpreted cautiously until replicated.

### 4.5. Mechanisms of Strength Loss and Relative Preservation of Explosive Force

The divergence between lower PF and largely comparable RFD in the upper limb of the PD group deserves consideration. Reduced maximal force in PD has been linked to central factors, including suboptimal motor unit firing rates and reduced voluntary activation of available muscle mass [15,16,17]. The observed association between PF and motor symptom severity is consistent with the possibility that these deficits become more evident as disease severity increases.

By contrast, the relative similarity of most RFD measures between groups may indicate that the neural drive required for the initial burst of force is less-affected than the capacity to achieve higher maximal force, at least in the upper limb and in mild-to-moderate PD. This interpretation is consistent with the idea that the scaling of force output and the timing of force generation may depend partly on distinct neural processes, with maximal force perhaps being more vulnerable in this context. Nevertheless, this explanation remains hypothetical and should be addressed in future studies using neurophysiological methods.

### 4.6. Clinical Implications

The fact that maximal handgrip PF emerged as a consistent correlate of gait speed and step length in both groups suggests that its clinical value in PD may lie more in reflecting general functional capacity than in serving as a specific marker of neurodegenerative progression. In clinical settings where comprehensive gait analysis is not feasible because of limitations in space, time, or equipment, handgrip dynamometry may provide a practical and low-cost indication of a patient’s general functional capacity. Accordingly, handgrip assessment should be regarded as a complementary screening or monitoring measure rather than a substitute for a comprehensive functional evaluation that includes the direct assessment of gait, balance, lower-limb strength, and other clinically relevant domains.

From a rehabilitation perspective, the shared pattern of associations across groups suggests that maintaining or improving overall force-generating capacity may be relevant for preserving mobility in aging and PD. Although task-specific gait training remains essential, the present findings support the inclusion of strength-oriented interventions as a complementary strategy to enhance neuromuscular reserve. In this context, handgrip strength should not be interpreted as a direct surrogate of lower-limb function, but rather as an accessible indicator of broader strength-related functional capacity.

### 4.7. Limitations and Future Directions

This study has several limitations that should be acknowledged. First, the cross-sectional design precludes causal inferences regarding the role of handgrip strength in gait impairment. Second, although the sample size was sufficient to detect moderate associations, it may have been insufficient to detect small associations involving RFD and impulse. Therefore, the absence of statistically significant associations for these variables should not be interpreted as definitive evidence of no relationship. Third, although handgrip PF may serve as a practical proxy of general functional capacity, it does not capture the specific contribution of lower-limb muscle groups directly involved in locomotion. Fourth, gait assessment was limited to preferred- and maximal-speed walking under relatively simple overground conditions. Consequently, we did not examine gait variability, dual-task interference, or dynamic balance, contexts in which explosive force may be more functionally relevant. Fifth, participants with PD were assessed in the ON-medication state, which may have attenuated some disease-related motor deficits. Finally, the large number of correlations examined increases the possibility of type I error.

In addition, the sample predominantly included individuals with mild-to-moderate PD, which may limit generalizability to more advanced disease stages or to specific clinical phenotypes, such as the postural instability/gait difficulty subtype. Future studies with larger cohorts are needed to validate these exploratory findings, provide more precise estimates of small associations, and strengthen the conclusions regarding the clinical relevance of RFD and impulse. In addition, future longitudinal studies should determine whether upper-limb RFD becomes more clinically informative as disease severity progresses or when motor tasks involve greater reactive or cognitive demands. Studies combining strength assessment with more challenging gait paradigms, and potentially with neurophysiological or neuroimaging approaches, may help clarify whether the handgrip–gait relationship reflects disease-specific mechanisms or broader systemic changes in functional capacity.

## 5. Conclusions

In summary, maximal handgrip strength, but not the upper-limb rate-of-force development or impulse variables, was associated with key gait outcomes, particularly gait speed and step length, in both individuals with PD and healthy older adults. This pattern suggests that the relationship between handgrip strength and gait may reflect general functional capacity rather than a PD-specific mechanism. Although individuals with PD showed lower maximal grip strength and poorer gait performance than controls, most upper-limb explosive force variables were broadly comparable between groups in this sample. These findings support the clinical utility of maximal handgrip strength as a practical indicator of general functional capacity and reinforce the rationale for including strength-oriented interventions as part of mobility-focused rehabilitation in older adults and individuals with PD.

## Figures and Tables

**Table 1:** Clinical characteristics of PD patients.

**Female**	23
**Male**	22
**MMSE**	23.62 ± 4.07
**Disease duration, years**	4.79 ± 3.84
**MDS-UPDRS-III**	27.67 ± 16.73
**MMSE**	23.62 ± 4.07
**H&Y stage (subject in each stage)**	1 (n = 14); 1.5 (n = 7); 2 (n = 10); 2.5 (n = 9); 3 (n = 5)
**Freezing of gait, n (%)**	12 (26.7%); predominantly mild/intermittent
**Clinical phenotype, n (%)**	Tremor-dominant: 20 (44.4%); indeterminate: 10 (22.2%); PIGD: 15 (33.3%)
**Levodopa-equivalent daily dose (mg/day)**	348 ± 266

Values are mean ± SD unless otherwise indicated. MMSE, Mini-Mental State Examination; MDS-UPDRS Part III, Movement Disorder Society–Unified Parkinson’s Disease Rating Scale, motor examination; PIGD, postural instability/gait difficulty.

**Table 2:** Comparison of characteristics and maximal strength between the Parkinson’s disease and healthy control groups.

	PD Group (n = 45)	HC Group (n = 51)	t (95% CI)	*p*-Value
Age (years)	60.73 ± 11.21	61.14 ± 10.70	−0.40 (−4.85, 4.04)	0.86
Body mass (kg)	72.33 ± 10.42	78.69 ± 13.62	−2.49 (−7.47, 2.5)	0.32
Height (cm)	166.20 ± 7.65	168.04 ± 8.9	−1.84 (−5.24, 1.56)	0.29
PF dominant (N)	235.13 ± 73.08	251.81 ± 86.53	−16.68 (−50.07, 16.72)	0.32
PF non-dominant (N)	210.88 ± 69	234.79 ± 78.01	−23.92 (−54.77, 6.94)	0.13

Data are presented as mean ± standard deviation. PD, Parkinson’s disease; HC, healthy controls; PF, peak force; CI, confidence interval. Independent-sample *t*-tests were used for between-group comparisons.

**Table 3:** Comparison of spatiotemporal gait parameters in the preferred- and maximal-speed conditions between groups.

	PD Group (n = 45)	HC Group (n = 51)	t (95% CI)	*p*-Value	Cohen’s d
***Preferred speed***					
Speed (m/s)	1.17 ± 0.27	1.32 ± 0.18	−3.22 (−2.4, −0.1)	0.002 *	0.66
Cadence (steps/min)	112.74 ± 14.02	114.04 ± 10.28	−0.52 (−6.24, 3.65)	0.6	0.11
Step length (m)	0.72 ± 0.11	0.80 ± 0.06	−3.94 (−0.11, −0.04)	<0.001 **	0.81
***Maximal speed***					
Speed (m/s)	1.71 ± 0.32	1.86 ± 0.28	−2.48 (−0.28, −0.03)	0.02	0.51
Cadence (steps/min)	136.09 ± 14	139.33 ± 13.78	−1.14 (−8.89, 2.39)	0.26	0.23
Step length (m)	0.87 ± 0.13	0.93 ± 0.1	−2.88 (−0.11, −0.02)	0.005 *	0.59

Data are presented as mean ± standard deviation. PD, Parkinson’s disease; HC, healthy controls; CI, confidence interval. The significance level was set at *p* < 0.008 after Bonferroni correction. * *p* < 0.008; ** *p* < 0.001.

## Data Availability

The data supporting the findings of this study are available from the corresponding author upon reasonable request. The data are not publicly available because they contain information that could compromise participant privacy.

## Abbreviations

The following abbreviations are used in this manuscript:

CI	Confidence interval
FOG	Freezing of gait
HC	Healthy controls
Imp	Force impulse
MDS-UPDRS	Movement Disorder Society-Unified Parkinson’s Disease Rating Scale
MIVC	Maximal isometric voluntary contraction
PD	Parkinson’s disease
PF	Peak force
RFD	Rate–of–force development
TFO	Time–to–force onset
TPF	Time–to–peak force
